# Mitochondria-Derived Superoxide Links to Tourniquet-Induced Apoptosis in Mouse Skeletal Muscle

**DOI:** 10.1371/journal.pone.0043410

**Published:** 2012-08-17

**Authors:** Thai P. Tran, Huiyin Tu, Jinxu Liu, Robert L. Muelleman, Yu-Long Li

**Affiliations:** 1 Department of Emergency Medicine, University of Nebraska Medical Center, Omaha, Nebraska, United States; 2 Department of Cellular and Integrative Physiology, University of Nebraska Medical Center, Omaha, Nebraska, United States; University of Cincinnati, United States of America

## Abstract

Our previous study has reported that superoxide mediates ischemia-reperfusion (IR)-induced necrosis in mouse skeletal muscle. However, it remains poorly understood whether IR induces apoptosis and what factors are involved in IR-induced apoptosis in skeletal muscle. Using a murine model of tourniquet-induced hindlimb IR, we investigated the relationship between mitochondrial dysfunction and apoptosis in skeletal muscle. Hindlimbs of C57/BL6 mice were subjected to 3 h ischemia and 4 h reperfusion via placement and release of a rubber tourniquet at the greater trochanter. Compared to sham treatment, tourniquet-induced IR significantly elevated mitochondria-derived superoxide production, activated opening of mitochondrial permeability transition pore (mPTP), and caused apoptosis in the gastrocnemius muscles. Pretreatment with a superoxide dismutase mimetic (tempol, 50 mg/kg) or a mitochondrial antioxidant (co-enzyme Q_10_, 50 mg/kg) not only decreased mitochondria-derived superoxide production, but also inhibited mPTP opening and apoptosis in the IR gastrocnemius muscles. Additionally, an inhibitor of mPTP (cyclosporine A, 50 mg/kg) also inhibited both mPTP opening and apoptosis in the IR gastrocnemius muscles. These results suggest that mitochondria-derived superoxide overproduction triggers the mPTP opening and subsequently causes apoptosis in tourniquet-induced hindlimb IR.

## Introduction

Exsanguinating injury of the extremity is a major cause of battlefield deaths and an important cause of preventable trauma fatalities in civilian medicine [1]–[6]. As an effective means of arresting life-threatening limb hemorrhage, tourniquet is commonly used in both civilian and battlefield settings [7]–[9]. However, stopping the blood flow in the traumatized limb with a tourniquet, and following reperfusion can cause the ischemia-reperfusion (IR) injury [10]. Therefore, an understanding of the pathomechanisms responsible for the tourniquet-induced IR injury can lead to novel therapeutic interventions to minimize the skeletal muscle IR injury induced by tourniquet.

The cell death secondary to IR is a mixture of cell necrosis and apoptosis [11], [12]. The major characteristics of necrosis are cell swelling and irreversible rupture of the plasma membrane [11], [12]. The major characteristics of apoptosis are cell shrinkage, DNA damage, chromatin condensation and fragmentation [11],[12]. Our previous study has shown that tourniquet-induced IR significantly causes cell necrosis (infarct size) in mouse gastrocnemius muscle; and superoxide overproduction and reduced antioxidant activity contribute to this IR injury [13]. Although apoptosis has been extensively investigated in many other tissues as a major trigger for IR-induced cell death [14]–[19], a few studies reported IR-induced apoptosis in skeletal muscle [20], [21]. More importantly, it is unclear whether tourniquet-induced IR can cause apoptosis and what mechanisms are involved in this type of cell death in the skeletal muscles. Using a model of tourniquet-induced acute murine hindlimb IR, therefore, our present study investigated IR-induced apoptosis and potential mechanisms responsible for the apoptosis of the skeletal muscles.

**Figure 1:**
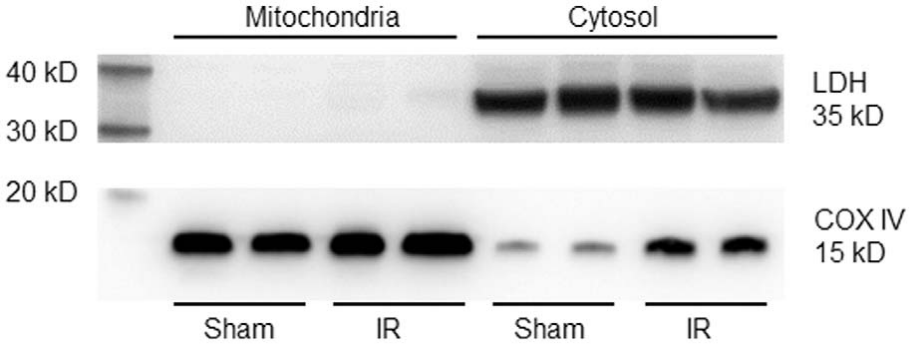
Evaluation of the purity of mitochondria isolated from the gastrocnemius muscles in sham and tourniquet-induced ischemia-reperfusion (IR) mice. LDH (lactate dehydrogenase, cytosolic protein maker) and COX IV (cytochrome c oxidase subunit IV, mitochondrial protein marker) were measured by Western blot analysis.

## Materials and Methods

### Animals

Male C57BL6 mice (10–12 weeks of age, 27–34 g, n = 102, Charles River Laboratory) were housed under controlled temperature and humidity and a 12∶12-h dark-light cycle, and were provided water and mouse chow *ad libitum.* Experiments were approved by the University of Nebraska Medical Center Institutional Animal Care and Use Committee and were carried out in accordance with the National Institutes of Health (NIH Publication No. 85-23, revised 1996).

### Drug Treatments

Mice were assigned randomly to sham and tourniquet-induced IR groups. In sham and IR groups, mice were intraperitoneally administered vehicle, 4-hydroxy-2,2,6,6-tetramethyl-piperidinyloxy (tempol, a superoxide dismutase mimetic, Alexis Biochemicals Co., CA), cyclosporine A (CsA, an inhibitor of mitochondrial permeability transition pore, Sigma-Aldrich, St.Louis, MO), or co-enzyme Q_10_ (CoQ_10_, a mitochondrial antioxidant, MP Biomedicals, OH), respectively. Vehicle, tempol (50 mg/kg), or CsA (50 mg/kg) was administered thirty minutes before tourniquet or sham procedure. For CoQ_10_ (50 mg/kg), mouse was intraperitoneally treated with CoQ_10_ at 24 h and 2 h before tourniquet, which based on the uptake and distribution of CoQ_10_ [13], [22].

**Figure 2:**
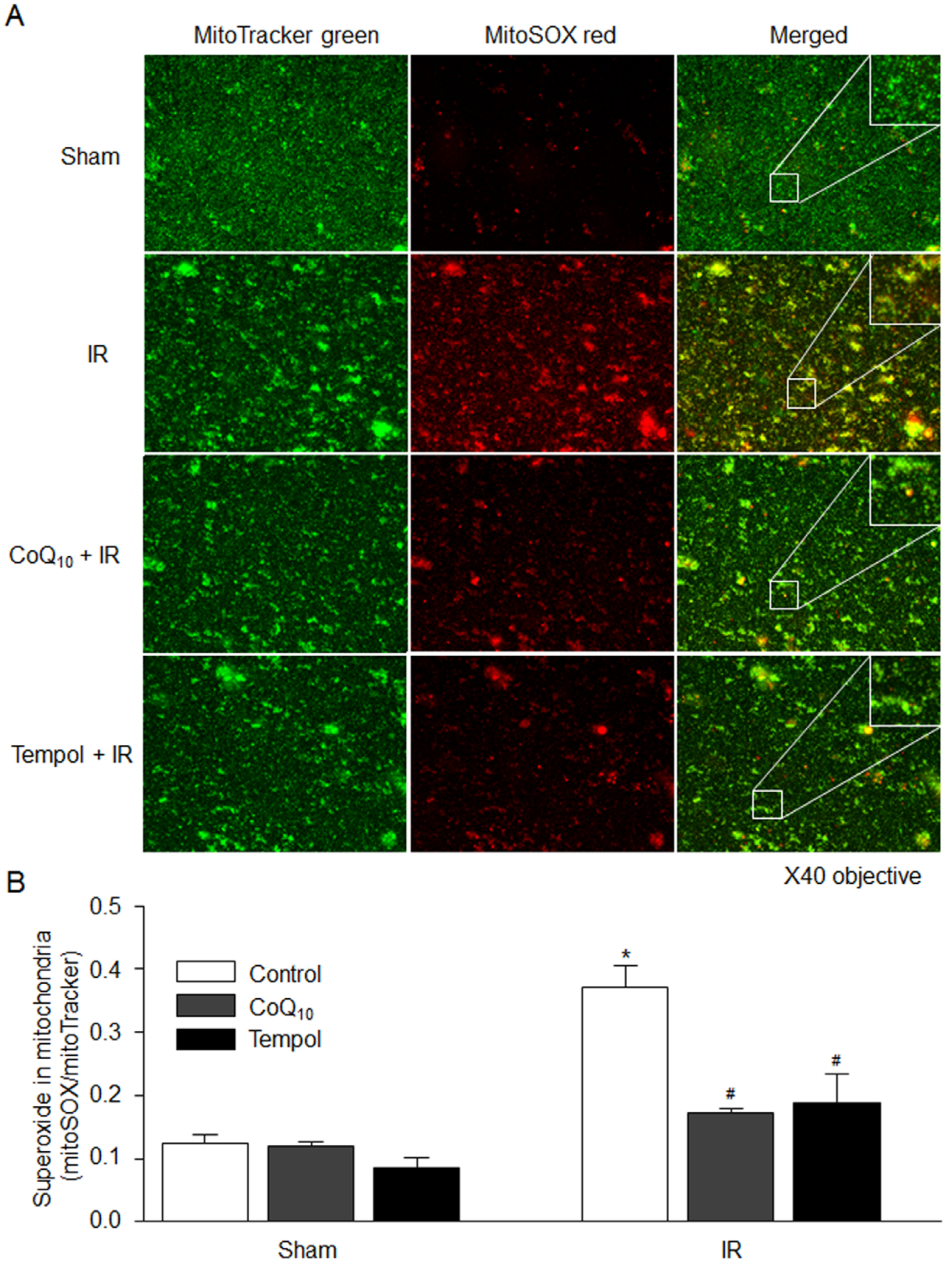
Mitochondria-derived superoxide production in the isolated mitochondria of the gastrocnemius muscles from each experimental group. MitoTracker green: mitochondrial marker; MitoSOX red: mitochondrial superoxide marker. Inset images were enlarged to ×100. Data are mean ± S.E.M., n = 6 mice in each group. *P<0.05 vs. sham control; ^#^p<0.05 vs. IR control.

**Figure 3:**
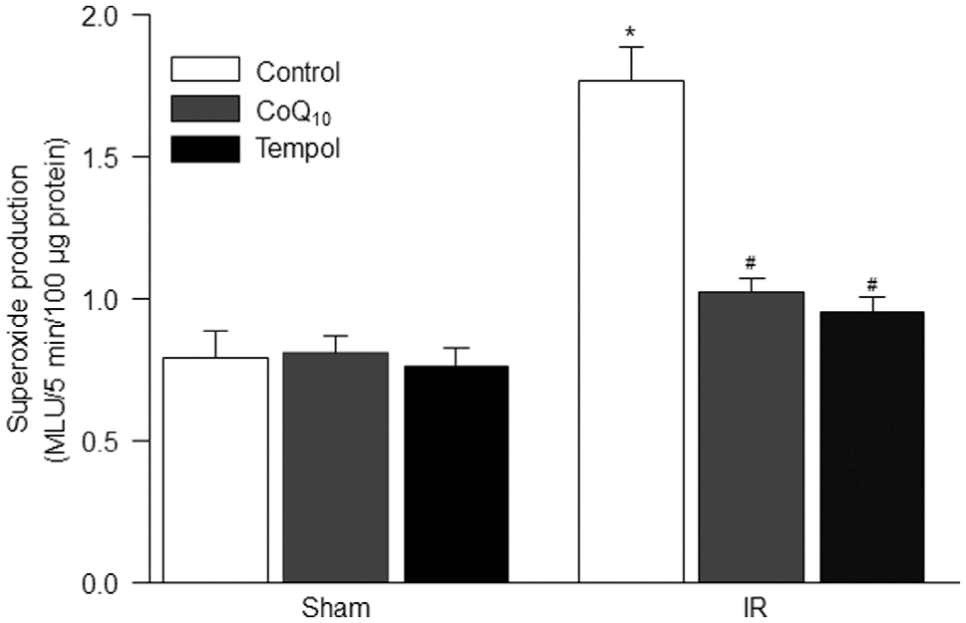
Mitochondria-derived superoxide production in the isolated mitochondria of the gastrocnemius muscles, measured by lucigenin chemiluminescent assay. Data are mean ± S.E.M., n = 6 mice in each group. *P<0.05 vs. sham control; ^#^p<0.05 vs. IR control.

### Acute Hindlimb IR Model

Mice were anesthetized with an anesthetic cocktail consisting of 0.1 mg/g ketamine and 0.01 mg/g xylazine, given as an intraperitoneal injection (0.01 ml/g body weight). The level of anesthesia was continuously monitored by observing the respiratory patterns and toe pinch reflex. Anesthesia was maintained throughout the duration of experiments with additional anesthetic cocktail (0.1 ml) as needed. The animals were restrained on a heating pad to maintain body temperature at 37°C.

**Figure 4:**
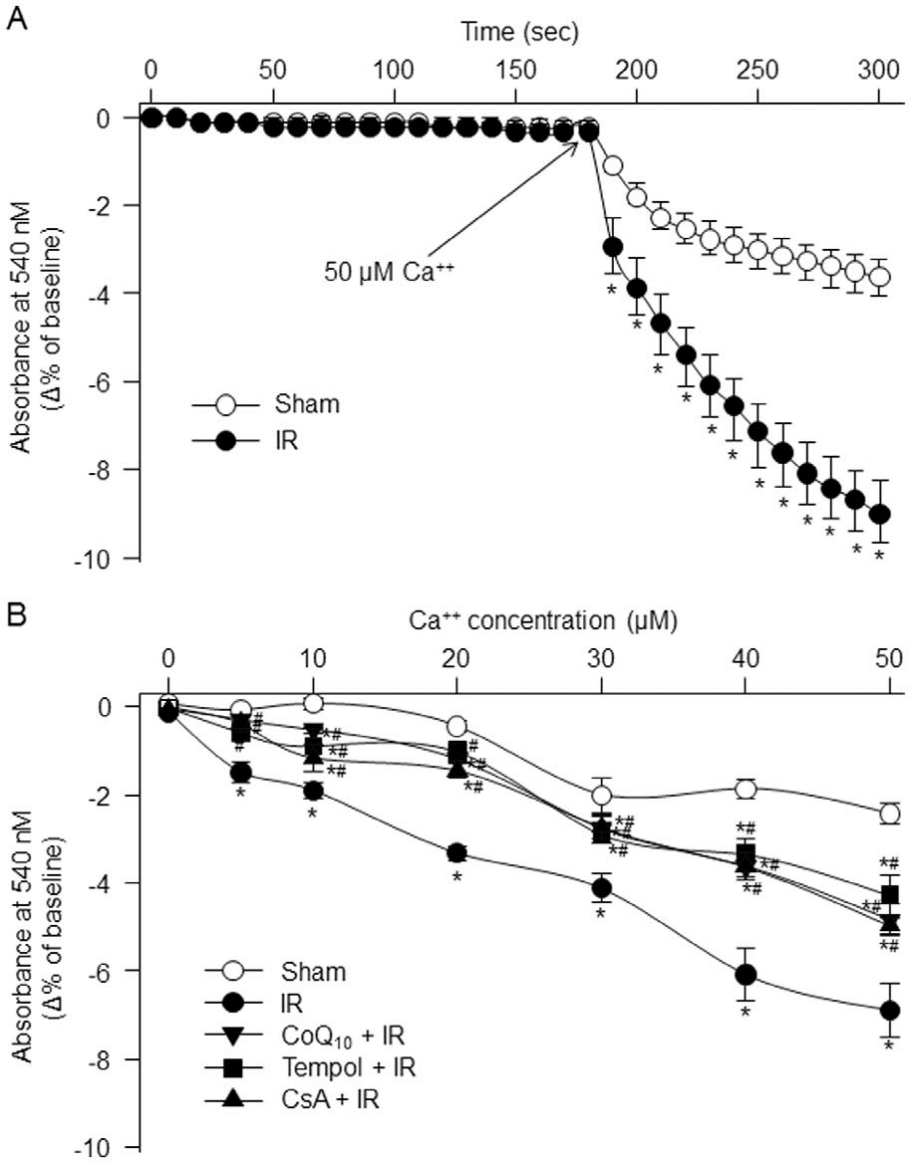
Calcium-induced mitochondrial swelling in the isolated mitochondria of the gastrocnemius muscles, measured by monitoring mitochondrial size at an absorbance of 540 nm. A, time-course for the changes of the absorbance at 540 nm in the isolated mitochondria of the gastrocnemius muscles from sham and tourniquet-induced IR groups. B, mitochondrial swelling induced by different calcium concentrations in the isolated mitochondria of the gastrocnemius muscles from each experimental group. Data are mean ± S.E.M., n = 7 mice in each group. *P<0.05 vs. sham; ^#^p<0.05 vs. IR.

Unilateral hind limb ischemia was induced by placing an orthodontic rubber band at the hip joint using a McGivney hemorrhoidal ligator [13], [23]. After 3 h ischemia, the orthodontic rubber band tourniquet was released and the hindlimb underwent 4 h reperfusion. Sham-operated animals were subjected to the same procedure except for the application of the orthodontic rubber band (i.e., no ischemia). During the entire procedure, mice were kept hydrated with intraperitoneal injection of 0.2 ml normal saline every 2 h. Tourniquet-induced IR was identified by measuring blood flow to the gastrocnemius muscle, as described previously [13]. Blood flow dropped to about 2% of baseline after placement of tourniquet and remained steady during 3 h ischemia. Upon tourniquet release, a rapid and transient increase in the blood flow to approximately 50% of baseline was observed, which was followed by a decline to a steady state of about 30% of baseline.

**Figure 5:**
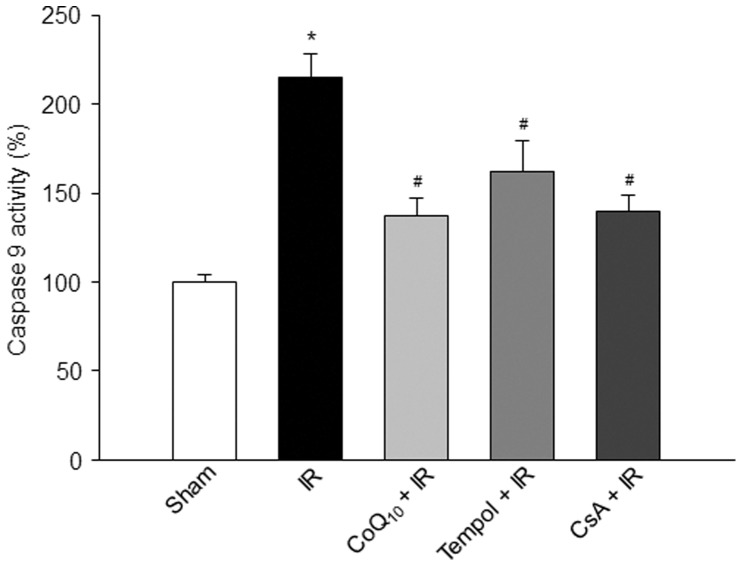
Caspase 9 activity in the gastrocnemius muscles from each experimental group. Data are mean ± S.E.M., n = 6 mice in each group. *P<0.05 vs. sham; ^#^p<0.05 vs. IR.

### Isolation of Mitochondria and Cytosol in Gastrocnemius Muscle

At the end of sham or tourniquet-induced IR protocol (3 h ischemia and 4 h reperfusion), gastrocnemius muscle was immediately harvested and washed with a cold isolation buffer A (in mM): 70 sucrose, 210 mannitol, 1 EDTA, 50 Tris-HCl (pH 7.4). After the adherent fascias, fats, and tendons were removed, the muscle was finely minced with scissors and then homogenized in the isolation buffer A using a Kontes tissue grinder. The homogenate was centrifuged at 1,300 g for 3 min. The supernatant was poured through cheesecloth and centrifuged at 10,000 g for 10 min. The supernatant was transferred into a fresh tube and kept on the ice as cytosolic fraction. The pellet was suspended in an isolation buffer B (in mM): 70 sucrose, 210 mannitol, 0.1 EDTA, 50 Tris-HCl (pH 7.4) and centrifuged at 6800 g for 10 min. The final pellets was diluted into an appropriate volume and kept on the ice as mitochondrial fraction. Mitochondrial or cytosolic protein concentration was determined using a bicinchoninic acid protein assay kit (Pierce; Rockford, IL). The fresh mitochondrial and cytosolic fractions were used for the following measurements.

**Figure 6:**
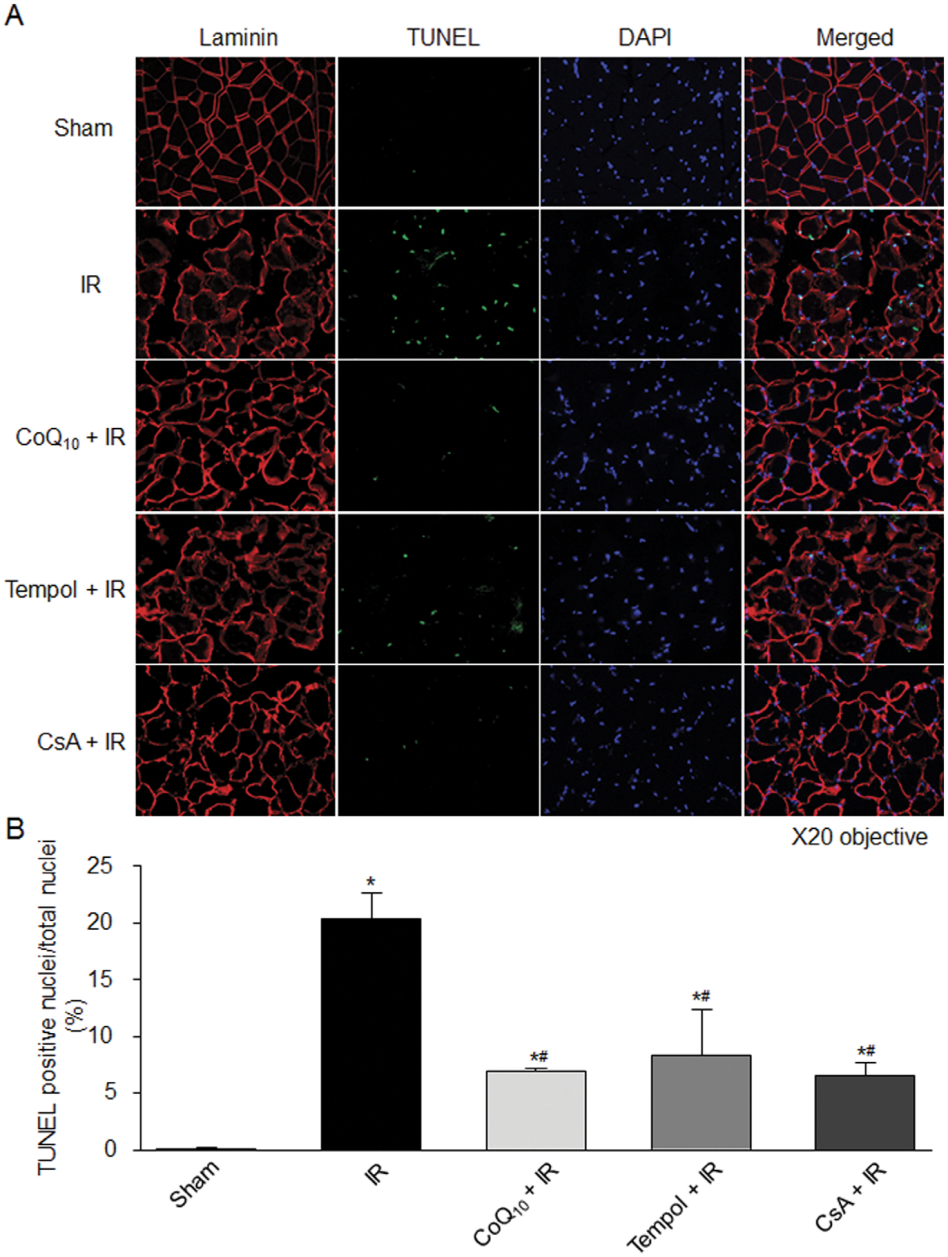
Apoptosis measured by TUNEL staining in the gastrocnemius muscles from each experimental group. Laminin: a marker for sarcolemma; TUNEL: terminal dUTP nick-end labeling, a marker for apoptosis; DAPI: a cell nucleus marker. Data are mean ± S.E.M., n = 5 mice (5 slices in each mouse) in each group. *P<0.05 vs. sham; ^#^p<0.05 vs. IR.

### Western Blot Analysis for Lactate Dehydrogenase (LDH) and Cytochrome C Oxidase IV (COX IV)

The sample of the isolated mitochondria or cytosol was mixed with loading buffer containing β-mercaptoethanol and heated at 100°C for 5 min. Equal amounts of the protein were loaded. Protein was fractionated in a 13% polyacrylamide gel along with molecular weight standards and transferred to PVDF membrane. The membrane was probed with rabbit anti-LDH (Santa Cruz Biotechnology, Santa Cruz, CA) or anti-COX IV (Abcam, Cambridge, MA) antibody and a peroxidase-conjugated goat anti-rabbit IgG (Pierce Chemical, Rockford, IL). The signal was detected using enhanced chemiluminescence substrate (Pierce Chemical, Rockford, IL) and the bands analyzed using UVP BioImaging Systems.

### In situ Detection of Mitochondrial Superoxide

Mitochondria-derived superoxide was measured using the mitochondria-targeted, superoxide-sensitive fluorogenic probe MitoSOX Red (Invitrogen), as described previously [24], [25]. Briefly, the fresh isolated mitochondria were loaded with MitoSOX Red (5 µM) and MitoTracker green (200 nM, a mitochondria marker; Invitrogen) at 36°C for 10 min. The images were captured using a Leica fluorescent microscope and quantified using Adobe Photoshop CS3 Extended (Adobe Systems).

### Lucigenin Chemiluminescent Assay for Superoxide Measurement

Superoxide anion production was measured in the isolated mitochondria of gastrocnemius muscles using the lucigenin chemiluminescence method described previously [13], [26]. The isolated mitochondria (0.2 ml) was placed in 0.5 ml microfuge containing dark–adapted lucigenin (5 µM) and NADH (100 µM), and then read in a TD-20/20 Luminometer (Turner Designs, Sunnyvale, CA). Light emission was recorded for 5 min and expressed as mean light units (MLU)/5 min/100 µg protein. Total protein concentration was determined using a bicinchoninic acid protein assay kit (Pierce; Rockford, IL).

### Ca^++^-induced Mitochondrial Permeability Transition Pore (mPTP) Opening [27]

The fresh isolated mitochondria (0.25 mg) are suspended in 0.5 ml reaction buffer (150 mM sucrose, 50 mM KCl, 2 mM KH_2_PO_4_, 5 mM succinic acid, 100 µM NADH, 5 mM HEPES with pH 7.4) at 36°C. After 3 min of equilibrium, different concentration of CaCl_2_ (0–50 µM) was added into the reaction buffer. The absorbance was continuously measured using a spectrophotometer (Genesys 6, Thermo Scientific, Waltham, MA) at 540 nm during 3 min of equilibrium and 2 min of calcium treatment. Ca^++^-induced decrease in absorbance was used to express the Ca^++^-induced mPTP opening.

### Measurement of Caspase 9 Activity

Caspase-9 colorimetric activity assay kit (EMD Millipore, Billerica, MA) was used to measure caspase-9 activity [28]. At the end of sham or tourniquet-induced IR protocol, gastrocnemius muscle was homogenized and centrifuged at 1,300 g for 3 min. The supernatant was added to assay mixture and incubated at 37°C for 2 h, and the absorbance at 405 nm wavelength was measured using Infinite M200 microplate reader (Tecan US, Durham, NC). According to the manufacturer’s instructions, caspase 9 activity (unit/mg protein) was calculated with a standard curve generated for each experiment.

### In situ Measurement of Terminal Deoxynucleotidyl Transferase-mediated dUTP Nick-end Labeling (TUNEL)

TUNEL was used to measure apoptosis of gastrocnemius muscles [29], [30]. At the end of sham or tourniquet-induced IR protocol, gastrocnemius muscles were frozen and cut into 10 µm-thick sections in a freezing cryostat at −20°C. To detect apoptotic DNA strand breaks, a fluorometric TUNEL detection kit was used according to the manufacturer’s instructions (Trevigen, Gaithersburg, MD). Briefly, muscle sections were fixed with 4% formaldehyde in PBS (pH 7.4) at room temperature for 20 min, permeabilized with protein K at room temperature for 30 min, and incubated with the labeling reaction mixture in a humidified chamber at 37°C for 1h. Pre-digestion of tissue section with nuclease was served as positive control. The sections were then processed with standard immunocytochemical staining procedure to incubate with antibody against laminin (a marker for sarcolemma; Sigma-Aldrich, St.Louis, MO) and DAPI (a cell nucleus marker; Invitrogen, Carlsbad, CA). Images were captured using a Leica fluorescent microscope, and the number of TUNEL and DAPI positive nuclei was counted. Percentage of TUNEL positive nuclei in total nuclei (DAPI positive nuclei) was used to express apoptosis.

### Data Analysis

All data are presented as mean ± SE. Sigmaplot 12 (Systat Software, Chicago, IL) was used for data analysis. A two-way ANOVA, with a Bonferroni procedure for *post hoc* was used in the comparison of all parameters. Normal distribution of data was confirmed with Kolmogorov-Smirnov test and equal variance with Levene’s test. Statistical significance was accepted when P<0.05.

## Results

### Purity of the Fresh Isolated Mitochondria

In order to measure the alterations of the mitochondrial functions (including superoxide production and mPTP opening), mitochondria of the gastrocnemius muscles were isolated and the purity of the fresh isolated mitochondria was evaluated by western blot analysis. As shown in Fig. 1, LDH (a cytosolic protein marker) significantly expressed in cytosolic fractions and was not detectable in mitochondrial fractions in sham and tourniquet-induced IR gastrocnemius muscles. This result demonstrates that mitochondria were purely isolated from the gastrocnemius muscles. COX IV (a mitochondrial protein marker) not only expressed in mitochondrial fractions but also mildly presented in cytosolic fractions. Additionally, expression of COX IV in cytosolic fractions from IR gastrocnemius muscles was higher than that from sham gastrocnemius muscles (Fig. 1). Furthermore, we also found that the content of mitochondria purely isolated from IR gastrocnemius muscles (0.098±0.007 mg mitochondrial protein/mg muscle protein, n = 13 mice) was lower than that from sham gastrocnemius muscles (0.180±0.013 mg mitochondrial protein/mg muscle protein, n = 13 mice, *p<0.05).

### Mitochondria-derived Superoxide Production in Gastrocnemius Muscles

Superoxide-sensitive probe MitoSOX Red is thought to be a useful tool for measuring mitochondria-derived superoxide [24], [25]. In Fig.2, tourniquet-induced IR significantly enhanced MitoSOX Red fluorescent images in the isolated mitochondria, which indicates an increase in mitochondria-derived superoxide in the gastrocnemius muscles. *In vivo* pretreatment of tempol (a superoxide dismutase mimetic) or CoQ_10_ (a mitochondrial antioxidant) markedly decreased mitochondria-derived superoxide production in the isolated mitochondria from tourniquet-induced IR gastrocnemius muscles but not sham gastrocnemius muscles.

We also investigated the effects of tempol and CoQ_10_ on mitochondria-derived superoxide production in the isolated mitochondria from sham and tourniquet-induced IR gastrocnemius muscles using lucigenin chemiluminescence method. Similarly, both tempol and CoQ_10_ normalized IR-enhanced mitochondria-derived superoxide production in the gastrocnemius muscles (Fig. 3). However, these chemicals did not affect the mitochondria-derived superoxide production in the isolated mitochondria from sham gastrocnemius muscles (Fig. 3).

### Ca^++^-induced mPTP Opening

Ca^++^-induced mPTP opening in isolated mitochondria was monitored by a decrease in 540 nm absorbance (Fig. 4). In mitochondria isolated from sham gastrocnemius muscles, 50 µM CaCl_2_ slightly induced the mPTP opening with time-dependent manner. The same concentration of CaCl_2_ significantly enhanced the mPTP opening in mitochondria isolated from IR gastrocnemius muscles, compared to sham gastrocnemius muscles (Fig. 4A). Additionally, the mPTP opening was markedly greater in mitochondria isolated from IR gastrocnemius muscles than from sham gastrocnemius muscles at all CaCl_2_ concentrations (5–50 µM) (Fig. 4B). Tempol, CoQ_10_, and cyclosporine A (CsA, an inhibitor of mPTP) each inhibited IR-enhanced mPTP opening (Fig. 4B). However, these chemicals did not influence Ca^2+^-induced mPTP opening in mitochondria isolated from sham gastrocnemius muscles (data not shown).

### Effects of Tempol, CoQ_10_, and CsA on Caspase 9 Activity in Sham and IR Gastrocnemius Muscles

A significant increase of caspase 9 activity was found in IR gastrocnemius muscles compared to sham gastrocnemius muscles (Fig. 5). *In vivo* pretreatment of tempol, CoQ_10_, or CsA markedly blunted IR-increased caspase 9 activity (Fig. 5); whereas they did not affect caspase 9 activity in sham gastrocnemius muscles (data not shown).

### Effects of Tempol, CoQ_10_, and CsA on TUNEL Staining in Sham and IR Gastrocnemius Muscles

TUNEL staining was performed to identify apoptotic nuclei (Fig. 6). We did not find TUNEL-positive nuclei in sham gastrocnemius muscles. Tourniquet-induced IR significantly increased the number of TUNEL-positive nuclei in gastrocnemius muscles (Fig. 6). Although tempol, CoQ_10_, and CsA did not alter the TUNEL-positive nuclei in sham gastrocnemius muscles, they partially decreased the number of TUNEL-positive nuclei in IR gastrocnemius muscles (Fig. 6).

## Discussion

Our present study reports that tourniquet-induced IR (3 h ischemia and 4 h reperfusion) increases mitochondria-derived superoxide production, causes mPTP opening, and induces apoptosis in mouse gastrocnemius muscles. A permeable superoxide dismutase mimetic (tempol) and a mitochondrial antioxidant (CoQ_10_) significantly inhibit IR-induced mitochondria-derived superoxide overproduction, mPTP opening, and apoptosis. Additionally, an inhibitor of mPTP (CsA) also blunts IR-induced mPTP opening and apoptosis. These results indicate that mitochondria-derived superoxide overproduction is involved in IR-induced apoptosis through promoting mPTP opening in mouse gastrocnemius muscles.

Apoptosis is a form of cell death, which is characterized by morphological and biochemical alterations including cell shrinkage, DNA damage, chromosomal condensation and fragmentation, and activation of caspases (caspase 3, 8, 9, etc.) [11], [12], [31]. Although apoptosis has been extensively investigated in many other tissues as a major trigger for IR-induced cell death [14]–[19], it is not clear whether apoptosis is involved in IR-induced cell death in skeletal muscle. The findings about IR-induced apoptosis in skeletal muscle are controversial. Using the primary cells isolated from rat skeletal muscles, Wang, et al. have demonstrated that IR causes both necrosis and apoptosis [20]. Using isolated rat spinotrapezius muscle preparation, Suzuki, et al. found that an increased incidence of DNA fragmentation occurred during IR [32]. However, in a rat lower limb tourniquet-induced IR model, two research groups [33], [34] did not find that TUNEL-positive nuclei appear in skeletal muscle during IR. Wang, et al. [20] think there are two possibilities to explain the different findings from above two research groups’ observations. First, the tourniquet might not completely stop arterial blood flow during ischemia, and might induce microcirculatory no-flow during reperfusion. Second, false positive or negative results of TUNEL staining sometimes make this technique unable to reflect cellular apoptosis in vivo. However, we do not think either of these is likely to occur in our present study. In the tourniquet-induced mouse IR model used in the present study, blood flow drops to about 2% of baseline during ischemia and recovers to approximately 30% of baseline during reperfusion in the gastrocnemius muscles [13]. Additionally, we combined a biochemical variable (caspase 9 activity) with TUNEL staining to evaluate apoptosis in all experiments. From our results, we found that tourniquet-induced IR increased the number of TUNEL-positive nuclei and caspase 9 activity in the gastrocnemius muscles (Figs. 5, 6), which clearly confirmed that tourniquet-induced IR causes apoptosis in skeletal muscle, besides necrosis [13].

Although the mechanisms responsible for IR-induced apoptosis are still unclear, much evidence has demonstrated that mitochondrial dysfunction could play a central role in cell death leading to both necrosis and apoptosis in many tissues [35]–[37]. In particular, mPTP may be involved in IR-induced cell death [38]. The mPTP is a non-selective channel to span the inner mitochondrial membrane and predominantly is in a closed state under the physiological condition. Although the role of the mPTP in healthy cells remains unclear, a major consequence of mPTP opening is found that the inner mitochondrial membrane no longer maintains a barrier to protons and all small molecular weight molecules, which dissipates the proton electrochemical gradient, inhibits ATP production, causes the mitochondrial swelling and rupture, finally initiates the apoptotic processes [39]–[43].In the present study, tourniquet-induced IR promoted mPTP opening (Fig. 4); and mPTP inhibitor (CsA) significantly inhibited IR-induced mPTP opening and subsequent apoptosis (Figs. 4, 5, 6). These data indicate that mPTP opening also mediates IR-induced cell apoptosis in the skeletal muscles.

Mitochondria as the major source of superoxide have been found in the skeletal muscle [44]–[47]. Although there has been considerable debate about the site(s) of superoxide generation within mitochondria, the most data indicate that complexes I and III of the electron transport chain are the main sites of mitochondrial superoxide production [47], [48]. Even if there is no information about role of superoxide in skeletal muscle mitochondrial dysfunction including mPTP opening, superoxide as a major factor contributes to mPTP opening in cardiac IR [49]. In the present study, mitochondria-derived superoxide was overproduced in the gastrocnemius muscles with tourniquet-induced IR (Figs. 1, 2). A permeable superoxide dismutase mimetic (tempol) and a mitochondrial antioxidant (CoQ_10_) markedly reduced mitochondria-derived superoxide overproduction, and inhibited mPTP opening and apoptosis in IR gastrocnemius muscles. Thus, mitochondria-derived superoxide is thought to be a major trigger for IR-induced apoptotic cell death in the skeletal muscles. Additionally, our present study clearly demonstrates that tempol, CoQ_10_, and CsA attenuate tourniquet-induced apoptosis in the skeletal muscles. However, we do realize that the protective roles of tempol, CoQ_10_, and CsA in muscle contractility and long-term limb functions need to be further confirmed in future *in vivo* studies.

There are several different types of cells in the skeletal muscles including skeletal myocytes, vascular endothelial cells, smooth muscle cells, infiltrated inflammatory cells, etc. In addition to skeletal myocytes, other types of cells might also produce superoxide and contribute to the apoptosis in skeletal muscle. However, compared to other types of cells, the skeletal myocytes are mitochondria-rich cells, which determines that the skeletal myocytes play a central role in mitochondria-derived superoxide production and apoptosis in the present study.

For the isolation of mitochondria, we found that content of pure mitochondria in IR gastrocnemius muscles was lower than that in sham gastrocnemius muscles. Additionally, we obtained mitochondria-contaminated cytosolic fraction even if pure mitochondrial fraction was isolated. One possibility is that mitochondria are broken by tissue homogenization and tourniquet-induced IR. The broken mitochondria could contaminate the cytosolic fraction in western blot analysis.

Recently, connexin-43, a major gap junction protein is thought to be involved in preconditioning-induced cardioprotection [50]–[56]. MPAK- and PKC-dependent mitochondrial translocation and phosphorylation of connexin-43 stimulate cardioprotection [53], [54], [56]. Connexin-43 inhibition with specific RNA interference significantly decreased cardiomyocyte survival [52], whereas mitochondria-specific overexpression of connexin-43 improved stem cell survival during heart cell therapy [51]. Additionally, phosphorylated connexin 43 inhibits the production of reactive oxygen species and mPTP opening [53]. Therefore, it is possible that alteration of connexin-43 also play a role in tourniquet-induced skeletal muscle apoptosis, which needs to be explored in our future studies.

In conclusion, mitochondria-derived superoxide is overproduced in skeletal muscle during tourniquet-induced IR. The elevation of mitochondria-derived superoxide contributes to IR-induced apoptosis in skeletal muscle through activating mPTP opening. These data further our understanding of the factors responsible for the tourniquet-induced apoptosis. More importantly, an improved understanding of the role of mitochondrial dysfunction in apoptosis may allow us to design effective therapeutic interventions and to improve tourniquet application in the civilian and battlefield setting.

## References

[1] Bellamy RF (1984) The causes of death in conventional land warfare: implications for combat casualty care research. Mil Med 149: 55–62. 6427656

[2] Carey ME (1996) Analysis of wounds incurred by U.S. Army Seventh Corps personnel treated in Corps hospitals during Operation Desert Storm, February 20 to March 10, 1991. J Trauma 40: S165–S169. 8606402 10.1097/00005373-199603001-00036

[3] Honda HM, Korge P, Weiss JN (2005) Mitochondria and ischemia/reperfusion injury. Ann N Y Acad Sci 1047: 248–258. 16093501 10.1196/annals.1341.022

[4] Mabry RL, Holcomb JB, Baker AM, Cloonan CC, Uhorchak JM, et al (2000) United States Army Rangers in Somalia: an analysis of combat casualties on an urban battlefield. J Trauma 49: 515–528. 11003332 10.1097/00005373-200009000-00021

[5] Mattox KL, Feliciano DV, Burch J, Beall AC Jr, Jordan GL Jr, et al (1989) Five thousand seven hundred sixty cardiovascular injuries in 4459 patients. Epidemiologic evolution 1958 to 1987. Ann Surg 209: 698–705. 2730182 10.1097/00000658-198906000-00007 PMC1494108

[6] Schmit-Neuerburg KP, Joka T (1985) Principles of treatment and indications for surgery in severe multiple trauma. Acta Chir Belg 85: 239–249. 4050256

[7] Beekley AC, Sebesta JA, Blackbourne LH, Herbert GS, Kauvar DS, et al (2008) Prehospital tourniquet use in Operation Iraqi Freedom: effect on hemorrhage control and outcomes. J Trauma 64: S28–S37. 18376169 10.1097/TA.0b013e318160937e

[8] Doyle GS, Taillac PP (2008) Tourniquets: a review of current use with proposals for expanded prehospital use. Prehosp Emerg Care 12: 241–256. 18379924 10.1080/10903120801907570

[9] Lee C, Porter KM, Hodgetts TJ (2007) Tourniquet use in the civilian prehospital setting. Emerg Med J 24: 584–587. 17652690 10.1136/emj.2007.046359 PMC2660095

[10] Blaisdell FW (2002) The pathophysiology of skeletal muscle ischemia and the reperfusion syndrome: a review. Cardiovasc Surg 10: 620–630. 12453699 10.1177/096721090201000620

[11] Majno G, Joris I (1995) Apoptosis, oncosis, and necrosis. An overview of cell death. Am J Pathol 146: 3–15. 7856735 PMC1870771

[12] Van CS, Van Den BW (2002) Morphological and biochemical aspects of apoptosis, oncosis and necrosis. Anat Histol Embryol 31: 214–223. 12196263 10.1046/j.1439-0264.2002.00398.x

[13] Tran TP, Tu H, Pipinos II, Muelleman RL, Albadawi H, et al (2011) Tourniquet-induced acute ischemia-reperfusion injury in mouse skeletal muscles: Involvement of superoxide. Eur J Pharmacol 650: 328–334. 21036124 10.1016/j.ejphar.2010.10.037 PMC3008320

[14] Lopez-Neblina F, Toledo AH, Toledo-Pereyra LH (2005) Molecular biology of apoptosis in ischemia and reperfusion. J Invest Surg 18: 335–350. 16319055 10.1080/08941930500328862

[15] Lee YM, Cheng PY, Chen SY, Chung MT, Sheu JR (2011) Wogonin suppresses arrhythmias, inflammatory responses, and apoptosis induced by myocardial ischemia/reperfusion in rats. J Cardiovasc Pharmacol 58: 133–142. 21436723 10.1097/FJC.0b013e31821a5078

[16] Rudiger HA, Clavien PA (2002) Tumor necrosis factor alpha, but not Fas, mediates hepatocellular apoptosis in the murine ischemic liver. Gastroenterology 122: 202–210. 11781294 10.1053/gast.2002.30304

[17] Li Y, Shen L, Cai L, Wang Q, Hou W, et al (2011) Spatial-temporal expression of NDRG2 in rat brain after focal cerebral ischemia and reperfusion. Brain Res 1382: 252–8. 21241684 10.1016/j.brainres.2011.01.023

[18] Lv M, Liu Y, Zhang J, Sun L, Liu Z, et al (2011) Roles of inflammation response in microglia cell through Toll-like receptors 2/interleukin-23/interleukin-17 pathway in cerebral ischemia/reperfusion injury. Neuroscience 176: 162–72. 21182899 10.1016/j.neuroscience.2010.11.066

[19] Linkermann A, Brasen JH, Himmerkus N, Liu S, Huber TB, et al.. (2012) Rip1 (Receptor-interacting protein kinase 1) mediates necroptosis and contributes to renal ischemia/reperfusion injury. Kidney Int 10. 10.1038/ki.2011.450 22237751

[20] Wang WZ, Fang XH, Stephenson LL, Khiabani KT, Zamboni WA (2008) Ischemia/reperfusion-induced necrosis and apoptosis in the cells isolated from rat skeletal muscle. J Orthop Res 26: 351–356. 17902174 10.1002/jor.20493

[21] Wang WZ, Baynosa RC, Zamboni WA (2011) Therapeutic interventions against reperfusion injury in skeletal muscle. J Surg Res 171: 175–182. 21920546 10.1016/j.jss.2011.07.015

[22] Miles MV (2007) The uptake and distribution of coenzyme Q10. Mitochondrion 7 Suppl: S72–S77 10.1016/j.mito.2007.02.012 17446143

[23] Crawford RS, Hashmi FF, Jones JE, Albadawi H, McCormack M, et al (2007) A novel model of acute murine hindlimb ischemia. Am J Physiol Heart Circ Physiol 292: H830–H837. 17012358 10.1152/ajpheart.00581.2006

[24] Tu H, Liu J, Zhu Z, Zhang L, Pipinos II, et al (2012) Mitochondria-derived superoxide and voltage-gated sodium channels in baroreceptor neurons from chronic heart-failure rats. J Neurophysiol 107: 591–602. 22072507 10.1152/jn.00754.2011 PMC3349624

[25] Yin JX, Yang RF, Li S, Renshaw AO, Li YL, et al (2010) Mitochondria-produced superoxide mediates angiotensin II-induced inhibition of neuronal potassium current. Am J Physiol Cell Physiol 298: C857–C865. 20089930 10.1152/ajpcell.00313.2009 PMC3115892

[26] Li YL, Gao L, Zucker IH, Schultz HD (2007) NADPH oxidase-derived superoxide anion mediates angiotensin II-enhanced carotid body chemoreceptor sensitivity in heart failure rabbits. Cardiovasc Res 75: 546–554. 17499230 10.1016/j.cardiores.2007.04.006 PMC2062532

[27] Bopassa JC, Ferrera R, Gateau-Roesch O, Couture-Lepetit E, Ovize M (2006) PI 3-kinase regulates the mitochondrial transition pore in controlled reperfusion and postconditioning. Cardiovasc Res 69: 178–185. 16216231 10.1016/j.cardiores.2005.07.014

[28] Yao H, Tang X, Shao X, Feng L, Wu N, et al (2007) Parthenolide protects human lens epithelial cells from oxidative stress-induced apoptosis via inhibition of activation of caspase-3 and caspase-9. Cell Res 17: 565–571. 17339884 10.1038/cr.2007.6

[29] Dalla LL, Ravara B, Angelini A, Rossini K, Sandri M, et al (2001) Beneficial effects on skeletal muscle of the angiotensin II type 1 receptor blocker irbesartan in experimental heart failure. Circulation 103: 2195–2200. 11331262 10.1161/01.cir.103.17.2195

[30] Libera LD, Zennaro R, Sandri M, Ambrosio GB, Vescovo G (1999) Apoptosis and atrophy in rat slow skeletal muscles in chronic heart failure. Am J Physiol 277: C982–C986. 10564091 10.1152/ajpcell.1999.277.5.C982

[31] Nicholson DW (1999) Caspase structure, proteolytic substrates, and function during apoptotic cell death. Cell Death Differ 6: 1028–1042. 10578171 10.1038/sj.cdd.4400598

[32] Suzuki H, Poole DC, Zweifach BW, Schmid-Schonbein GW (1995) Temporal correlation between maximum tetanic force and cell death in postischemic rat skeletal muscle. J Clin Invest 96: 2892–2897. 8675660 10.1172/JCI118360 PMC186000

[33] Cowled PA, Leonardos L, Millard SH, Fitridge RA (2001) Apoptotic cell death makes a minor contribution to reperfusion injury in skeletal muscle in the rat. Eur J Vasc Endovasc Surg 21: 28–34. 11170874 10.1053/ejvs.2000.1209

[34] Knight KR, Messina A, Hurley JV, Zhang B, Morrison WA, et al (1999) Muscle cells become necrotic rather than apoptotic during reperfusion of ischaemic skeletal muscle. Int J Exp Pathol 80: 169–175. 10469272 10.1046/j.1365-2613.1999.00111.x PMC2517767

[35] Kroemer G, Reed JC (2000) Mitochondrial control of cell death. Nat Med 6: 513–519. 10802706 10.1038/74994

[36] Matsuyama S, Reed JC (2000) Mitochondria-dependent apoptosis and cellular pH regulation. Cell Death Differ 7: 1155–1165. 11175252 10.1038/sj.cdd.4400779

[37] Green DR, Reed JC (1998) Mitochondria and apoptosis. Science 281: 1309–1312. 9721092 10.1126/science.281.5381.1309

[38] Baines CP (2009) The mitochondrial permeability transition pore and ischemia-reperfusion injury. Basic Res Cardiol 104: 181–188. 19242640 10.1007/s00395-009-0004-8 PMC2671061

[39] Di LF, Canton M, Menabo R, Kaludercic N, Bernardi P (2007) Mitochondria and cardioprotection. Heart Fail Rev 12: 249–260. 17516167 10.1007/s10741-007-9028-z

[40] Halestrap AP, Clarke SJ, Javadov SA (2004) Mitochondrial permeability transition pore opening during myocardial reperfusion–a target for cardioprotection. Cardiovasc Res 61: 372–385. 14962470 10.1016/S0008-6363(03)00533-9

[41] Kroemer G (2003) The mitochondrial permeability transition pore complex as a pharmacological target. An introduction. Curr Med Chem 10: 1469–1472. 12905994 10.2174/0929867033457232

[42] Leung AW, Halestrap AP (2008) Recent progress in elucidating the molecular mechanism of the mitochondrial permeability transition pore. Biochim Biophys Acta 1777: 946–952. 18407825 10.1016/j.bbabio.2008.03.009

[43] Weiss JN, Korge P, Honda HM, Ping P (2003) Role of the mitochondrial permeability transition in myocardial disease. Circ Res 93: 292–301. 12933700 10.1161/01.RES.0000087542.26971.D4

[44] Lambert AJ, Buckingham JA, Brand MD (2008) Dissociation of superoxide production by mitochondrial complex I from NAD(P)H redox state. FEBS Lett 582: 1711–1714. 18442479 10.1016/j.febslet.2008.04.030

[45] Lambert AJ, Brand MD (2004) Superoxide production by NADH:ubiquinone oxidoreductase (complex I) depends on the pH gradient across the mitochondrial inner membrane. Biochem J 382: 511–517. 15175007 10.1042/BJ20040485 PMC1133807

[46] St-Pierre J, Buckingham JA, Roebuck SJ, Brand MD (2002) Topology of superoxide production from different sites in the mitochondrial electron transport chain. J Biol Chem 277: 44784–44790. 12237311 10.1074/jbc.M207217200

[47] Miller FL, Liu Y, Van Remmen H (2004) Complex III releases superoxide to both sides of the inner mitochondrial membrane. J Biol Chem 279: 49064–49073. 15317809 10.1074/jbc.M407715200

[48] Boveris A, Chance B (1973) The mitochondrial generation of hydrogen peroxide. General properties and effect of hyperbaric oxygen. Biochem J 134: 707–716. 4749271 10.1042/bj1340707 PMC1177867

[49] Murphy E, Steenbergen C (2008) Mechanisms underlying acute protection from cardiac ischemia-reperfusion injury. Physiol Rev 88: 581–609. 18391174 10.1152/physrev.00024.2007 PMC3199571

[50] Lu G, Ashraf M, Haider KH (2012) Insulin-like growth factor-1 preconditioning accentuates intrinsic survival mechanism in stem cells to resist ischemic injury by orchestrating protein kinase calpha-erk1/2 activation. Antioxid Redox Signal 16: 217–227. 21923556 10.1089/ars.2011.4112 PMC3263485

[51] Lu G, Jiang S, Ashraf M, Haider KH (2012) Subcellular preconditioning of stem cells: mito-Cx43 gene targeting is cytoprotective via shift of mitochondrial Bak and Bcl-xL balance. Regen Med 7: 323–334. 22594326 10.2217/rme.12.13 PMC3380626

[52] Lu G, Haider HK, Jiang S, Ashraf M (2009) Sca-1+ stem cell survival and engraftment in the infarcted heart: dual role for preconditioning-induced connexin-43. Circulation 19 119: 2587–2596. 10.1161/CIRCULATIONAHA.108.827691 PMC2839162 19414636

[53] Halestrap AP (2006) Mitochondria and preconditioning: a connexin connection? Circ Res 99: 10–12. 16825585 10.1161/01.RES.0000233145.94073.b8

[54] Rodriguez-Sinovas A, Boengler K, Cabestrero A, Gres P, Morente M, et al (2006) Translocation of connexin 43 to the inner mitochondrial membrane of cardiomyocytes through the heat shock protein 90-dependent TOM pathway and its importance for cardioprotection. Circ Res 99: 93–101. 16741159 10.1161/01.RES.0000230315.56904.de

[55] Boengler K, Schulz R, Heusch G (2006) Connexin 43 signalling and cardioprotection. Heart 92: 1724–1727. 16387816 10.1136/hrt.2005.066878 PMC1861299

[56] Lu G, Haider HK, Porollo A, Ashraf M (2010) Mitochondria-specific transgenic overexpression of connexin-43 simulates preconditioning-induced cytoprotection of stem cells. Cardiovasc Res 88: 277–286. 20833648 10.1093/cvr/cvq293 PMC2952537

